# PD-L1 Expression on Retrovirus-Infected Cells Mediates Immune Escape from CD8^+^ T Cell Killing

**DOI:** 10.1371/journal.ppat.1005224

**Published:** 2015-10-20

**Authors:** Ilseyar Akhmetzyanova, Malgorzata Drabczyk, C. Preston Neff, Kathrin Gibbert, Kirsten K. Dietze, Tanja Werner, Jia Liu, Lieping Chen, Karl S. Lang, Brent E. Palmer, Ulf Dittmer, Gennadiy Zelinskyy

**Affiliations:** 1 Institute for Virology, University Hospital Essen, University of Duisburg-Essen, Essen, Germany; 2 University of Colorado, Anschutz Medical Campus, Aurora, Colorado, United States of America; 3 Department of Infectious Diseases, Union Hospital of Tonji Medical College, Huazhong University of Science and Technology, Wuhan, P.R. China; 4 Department of Immunobiology, Yale School of Medicine, Yale University, New Haven, Connecticut, United States of America; 5 Institute for Immunology, University Hospital Essen, University of Duisburg-Essen, Essen, Germany; University of Pennsylvania, UNITED STATES

## Abstract

Cytotoxic CD8+ T Lymphocytes (CTL) efficiently control acute virus infections but can become exhausted when a chronic infection develops. Signaling of the inhibitory receptor PD-1 is an important mechanism for the development of virus-specific CD8+ T cell dysfunction. However, it has recently been shown that during the initial phase of infection virus-specific CD8+ T cells express high levels of PD-1, but are fully competent in producing cytokines and killing virus-infected target cells. To better understand the role of the PD-1 signaling pathway in CD8+ T cell cytotoxicity during acute viral infections we analyzed the expression of the ligand on retrovirus-infected cells targeted by CTLs. We observed increased levels of PD-L1 expression after infection of cells with the murine Friend retrovirus (FV) or with HIV. In FV infected mice, virus-specific CTLs efficiently eliminated infected target cells that expressed low levels of PD-L1 or that were deficient for PD-L1 but the population of PD-L1high cells escaped elimination and formed a reservoir for chronic FV replication. Infected cells with high PD-L1 expression mediated a negative feedback on CD8+ T cells and inhibited their expansion and cytotoxic functions. These findings provide evidence for a novel immune escape mechanism during acute retroviral infection based on PD-L1 expression levels on virus infected target cells.

## Author Summary

Virus-specific cytotoxic T cells can eliminate infected cells during acute viral infections, but in chronic infections these cells often become dysfunctional or “exhausted.” The inhibitory receptor PD-1 is involved in the suppression of cytotoxic T cell responses in chronic infections. However, during many acute viral infections cytotoxic T cells up-regulate the PD-1 receptor but initially remain competent in killing virus infected target cells. Here we show that the ligand for PD-1, called PD-L1, can be induced on retrovirus infected cells and that the cells with the highest expression of PD-L1 escaped from cytotoxic T cell killing. Thus, PD-L1high infected target cells accumulated during the course of infection, formed the reservoir of virus persistence, and subsequently mediated a negative feedback on cytotoxic T cells via the PD-1 receptor that ultimately resulted in functional exhaustion of these cells. The current results provide evidence for a novel escape mechanism of viruses from cytotoxic T cell responses and may explain how viral reservoirs are established during chronic infections.

## Introduction

Cytotoxic CD8^+^ T Lymphocytes (CTL) are crucial for controlling viruses and tumors. However, in several chronic viral infections, such as Human Immunodeficiency virus (HIV) and Hepatitis C virus (HCV) infection of humans or Lymphocytic Choriomeningitis virus (LCMV) and Friend virus (FV) infection of mice, virus-specific CD8^+^ T cells become functionally exhausted with ongoing infection. This exhaustion likely contributes to the inability of the host to eliminate cells infected with the pathogen [1, 2]. One of the mechanisms that leads to CD8^+^ T cell dysfunction is the signaling of the inhibitory receptor programmed death 1 (PD-1) that induces T cell exhaustion [3–5]. Blocking the interaction of this receptor-and its primary ligand, PD-L1, partially restores T cell function and reduces viral loads in chronically infected animals [3, 6–8]. PD-L1 is broadly expressed on different cells and organs while the other ligand for PD-1, PD-L2, is preferentially expressed on antigen presenting cells (APC). It has been shown in recent studies that effector T cells already up-regulate PD-1 during the acute phase of infection before virus becomes persistent or latent. This has been shown for infections of humans with Epstein Barr virus (EBV) [9], Hepatitis C virus (HCV) [10], or Hepatitis B virus (HBV) [11] as well as in monkeys infected with Simian Immunodeficiency virus (SIV) [12], and SIV-HIV hybrid virus (SHIV) [13]. Furthermore, the SIV study provides evidence that T cell receptor stimulation itself induces PD-1 expression on CD8^+^ T cells [12]. Activated CD8^+^ T cells up-regulate the expression of PD-1 but remain fully functional during the first two weeks of FV infection [14]. Thus, the appearance of PD-1 on effector CD8^+^ T cells does not per se induce the exhaustion of these cells. This suggests that the expression of the ligands for PD-1 might critically contribute to the functional involvement of PD-1 signaling in the development of viral chronicity. Interestingly, several therapeutic studies that target the PD-1/PD-L1 pathway to improve CTL functions during chronic infections or cancer utilize blocking antibodies against PD-L1 rather than PD-1 [15], but the regulation of PD-L1 expression and its functional relevance for CTL killing is less well understood than that of PD-1. APC and infected target cells are the main cell populations, which specifically interact with CTLs via immunologic synapses and have direct and long-lasting contacts with inhibitory receptors on the surface of CTLs. Thus, the expression of ligands for PD-1 on infected cells and APCs may be the key regulatory factor influencing the functionality of PD-1 expressing CD8^+^ T cells during acute as well as chronic infections. Different studies have demonstrated enhanced expression of inhibitory ligands in virus-infected organs. As an example, cardiac myocytes express significantly more PD-L1 after infection with Coxsackievirus B3, and the interaction of PD-L1 with PD-1 is critical for the development of cardiac myositis [16]. Mouse tissue infected with the Rabies virus (RV) or with LCMV also expressed enhanced levels of PD-L1 [17, 18]. Similarly, an *in vitro* study shows that respiratory syncytial virus (RSV) infected primary human bronchial endothelial cells expressed enhanced levels of PD-L1 and co-cultivation with effector CD8^+^ T cells resulted in decreased production of cytokines and cytotoxic molecules in the CD8^+^ T cells [19]. In addition, studies on HIV-1 infection of human macrophages in cell culture showed enhanced expression of PD-L1 and PD-L2 on the surface of infected cells [20]. However, the *in vivo* regulation of PD-L1 expression during an ongoing infection and its effect on CTL killing of virus-infected target cells has not been studied until now.

In the current study, the murine Friend retrovirus model was used to characterize the role of inhibitory ligand expression on FV infected cells in CTL immune escape during an acute retrovirus infection. FV is an oncogenic retroviral complex that can induce erythroleukemia in susceptible mice. However, resistant mouse strains, like the C57Bl/6 mice that we used in this study, mount a potent anti-viral immune response during the acute phase of infection that prevents the onset of leukemia [21]. Despite this efficient initial viral immunity, FV eventually escapes from T cell mediated immune control and establishes a chronic infection [22]. Interestingly, previous studies have shown that B cells and monocytes are the main reservoir for FV during chronic infection [23], but it is not known how these cells escape CTL destruction during the initial phase of infection. FV infected cells express the viral envelope protein gp70 on the cell membrane [24], which provides a unique method to detect infected cells ex vivo by surface staining with a specific antibody [25]. This allows these cells to be used for the kinetic analysis of PD-L1 expression levels. We observed that in vivo same populations of FV infected cells expressed high levels of PD-L1 and escaped elimination by CTLs. In addition, they contributed to the development of exhaustion of virus specific PD-1 expressing CD8^+^ T cells. The current study provides new and important data about the functional role of PD-L1 during acute retroviral infection and the involvement of inhibitory ligands in viral immune escape.

## Results

### PD-L1 expression levels on FV-infected target cells correlate with survival

**Fig 1:**
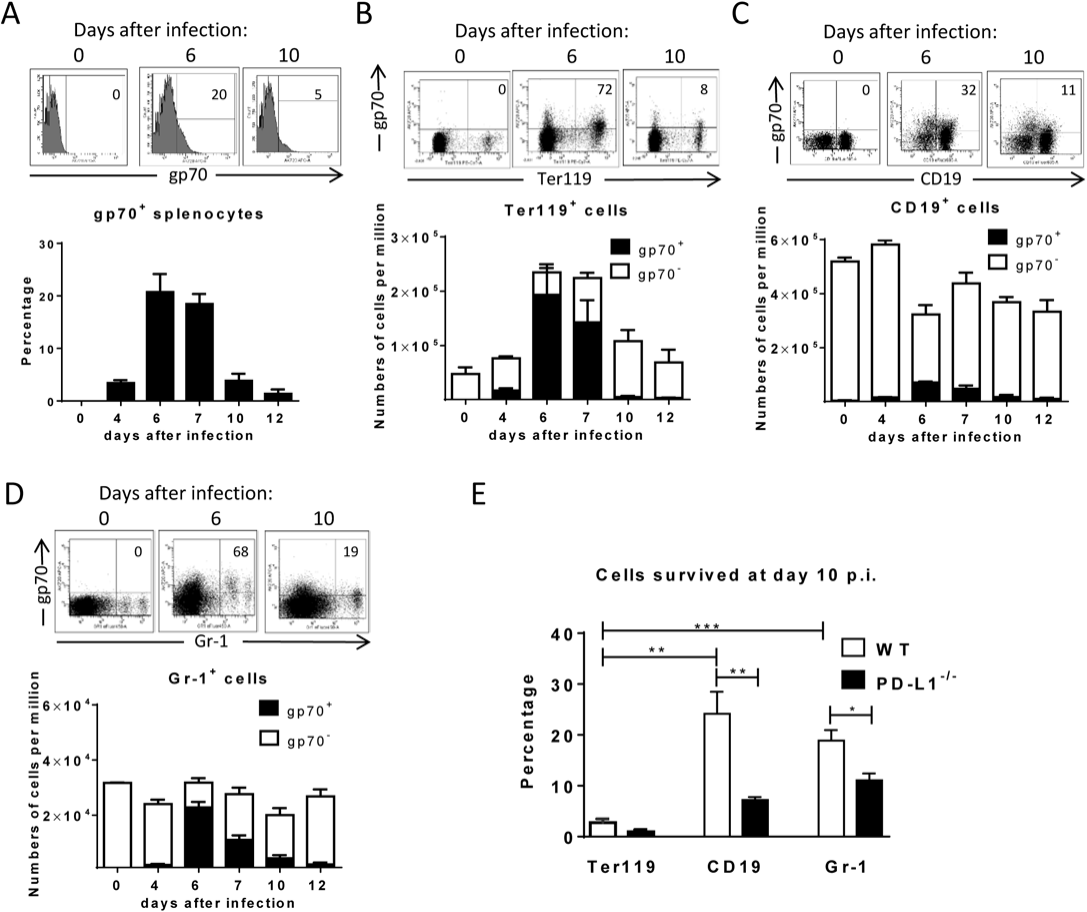
Target cell populations of FV infection. C57BL/6 mice were infected with FV and splenocytes were isolated at different time points after infection. Multi-parameter flow cytometry analysis was used to compare the expression of FV gp70 antigen on the cell surface of different subpopulations of spleen cells. **A**. representative histograms of all nucleated cells positive for gp70 from naïve, 6 day and 10 day infected mice. The bars represent percentages of all nucleated spleen cells positive for gp70 for a group of 6–10 mice. **B**. The representative dot plots of Ter119^+^ cells positive for gp70 from naïve, 6 day and 10 day infected mice. Numbers in the upper right quadrat represent the percentage of gp70^+^ of Ter119^+^ cells. The bars represent the number of non-infected Ter119^+^ erythroblasts (gp70 negative) per one million nucleated cells (white columns) and the number of infected Ter119^+^gp70^+^ cells (black columns). **C**. The representative dot plot of CD19^+^ cells positives for gp70 from naive, 6 day and 10 day infected mice. Numbers in the upper right quadrat represent the percentage of gp70^+^ of CD19^+^ cells. The bars represents the number of non-infected CD19^+^ B cells (gp70 negative) per one million nucleated cells (white columns) and the number of infected CD19^+^gp70^+^ cells (black columns). **D**. The representative dot plot of Gr-1^+^ cells positives for gp70 from naive, 6 day and 10 day infected mice. Numbers in the upper right quadrat represent the percentage of gp70^+^ of Gr-1^+^ cells. The bars represents the number of non-infected myeloid Gr-1^+^ cells (white column) and the number of infected Gr-1^+^ gp70^+^ cells (black columns). **E**. The frequency of gp70^+^ cells at day 10 post infection in relation to the infected cells at day 6 from C57BL/6 (wt) (white bars) and PD-L1^-/-^ (black bars) mice. Mean numbers plus SD from experiments with 5–8 mice are shown. Data was pooled from three independent experiments with similar results. Differences between frequencies of infected (gp70^+^) cells from different populations were analyzed by an unpaired t-test and are indicated in the figure (**p˂0.005, ***p˂0.0005).

The aim of this study was to determine *ex vivo* the expression of PD-L1 on FV infected cells during the acute phase (first two weeks) of infection. To perform such an analysis we first had to define the kinetics of FV infection in different target cell populations. It was known from previous studies that FV mainly replicates in Ter119^+^ erythroid precursor cells, CD19^+^ B cells and Gr-1^+^ myeloid cells [23], thus we focused our analysis on these three cell populations. Flow cytometry was used for the detection of FV envelope protein gp70 expressing cells recognized by antibody 720 (Ab720) [25]. Infected gp70 positive cells were detectible from day 4 after infection and peak gp70 expression on total splenocytes was observed at day 6 after infection (Fig 1A). This data confirms previous studies with an infectious center assay [26, 27]. On day 7 the percentage of infected gp70^+^ cells dropped, which correlated with the expansion of FV-specific cytotoxic CD8^+^ T cells [26]. The phenotypic characterization of cells expressing gp70 revealed that the main target population for the virus was Ter119^+^ cells (Fig 1B). FV induces the proliferation of Ter119^+^ erythroblasts via EPO receptor signaling and this population of expanded cells is the main reservoir for FV during the acute phase of infection [28]. Thus, the numbers of infected gp70^+^Ter119^+^ cells expanded dramatically until day 6 post infection, but after that time point most infected cells rapidly disappeared and almost no infection of Ter119^+^ cells could be detected anymore after 10 days. FV infection of B cells and Gr-1^+^ cells also peaked at 6 days post infection but the total numbers of infected cells were much lower than those of Ter119^+^ cells (Fig 1C and Fig 1D). Interestingly, the frequency of infected CD19^+^ and Gr-1^+^ cells also declined after day 6, but this decline was not as dramatic as observed for the Ter119^+^ target cells. To characterize this difference, we calculated the percentage of infected cells that had survived at day 10 compared to the peak infection at day 6 (Fig 1E; white bars). Whereas only 3% of the pool of FV infected Ter119^+^ cells were left at day 10 post infection, 25% of the infected B cells and 19% of the infected Gr-1^+^ myeloid cells were still detectable. This analysis clearly shows a preferential survival of FV infected B cells and Gr-1^+^ myeloid cells compared to erythroblasts. Since the control of FV replication during acute infection is dependent on killing of infected cells by virus-specific cytotoxic CD8^+^ T cells [26, 29], it is reasonable to believe that differences in the efficacy of CTL killing of distinct populations of infected target cells may exist.

**Fig 2:**
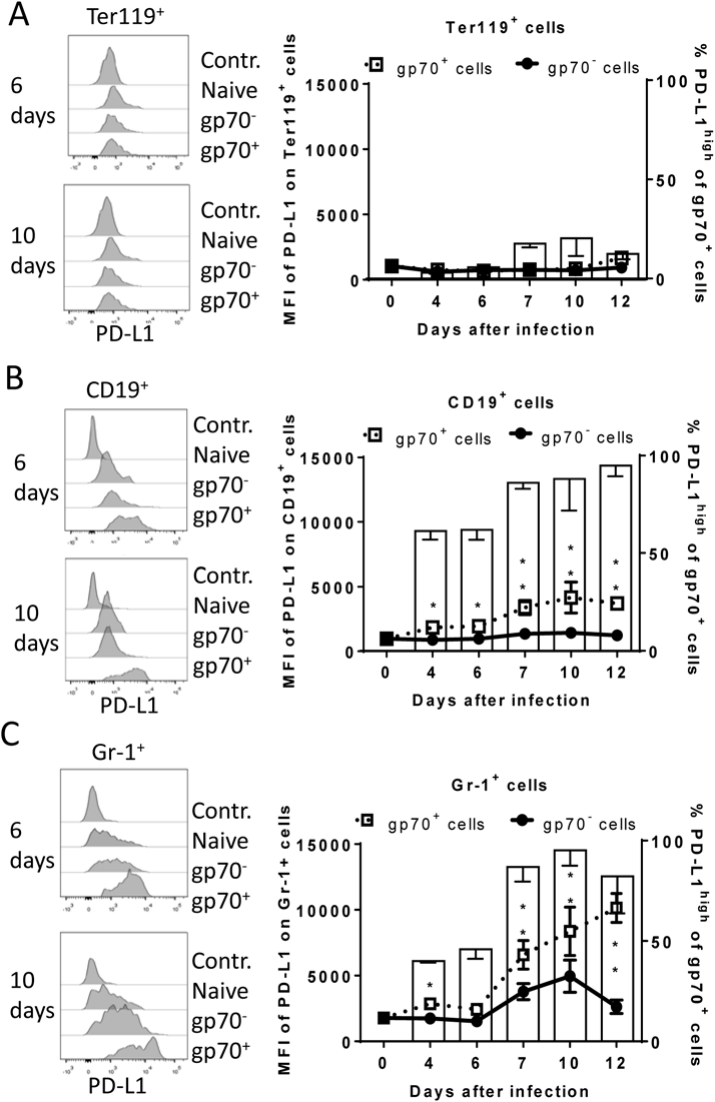
Expression of PD-L1 on the surface of FV infected cells. C57BL/6 mice were infected with FV and the splenocytes were isolated at different time points after infection. Multi-parameter flow cytometry was used to compare the expression (MFI) of PD-L1 on the cell surface of infected (gp70^+^) and non-infected (gp70^-^) Ter119^+^ erythroid precursor cells (**A**), CD19^+^ B cells (**B**), and Gr-1^+^ myeloid derived cells (**C**) and the percentage of PD-L1^high^ (white bars) of gp70^+^ cells. Data were pooled from three independent experiments with similar results. Representative histograms of PD-L1 expression on infected (gp70^+^) and non-infected (gp70^-^) cells gated on every analyzed cell population on day 6 and day10 in infected mice are shown. Mean numbers plus SD from experiments with 5–8 mice are shown. Data was pooled from three independent experiments with similar results. Differences between infected (gp70^+^) and non-infected (gp70^-^) cells were analyzed by an unpaired t-test and are indicated in the figure (*p˂0.05, **p˂0.005, ***p˂0.0005).

During acute FV infection virus-specific cytotoxic CD8^+^ T cells express high levels of PD-1 [14] leading us to deduce that the PD-1/PD-L1 pathway may be involved in escape of FV infected cells from CTL killing. We therefore analyzed expression levels of PD-L1 (MFI) on FV infected (gp70^+^) versus non-infected (gp70^-^) cell subsets and frequencies of PD-L1^high^ FV infected cells *ex vivo* at different time points during acute FV infection. Since the basic levels of PD-L1 expression on the three cell populations were different, we defined PD-L1^high^, as expression levels which were higher than on the same cell population in naïve mice (see Fig 2A–2C histograms). The population of Ter119^+^ cells did not significantly change in their intensity of PD-L1 expression or in percentages of PD-L1^high^ cells after FV infection (Fig 2A). In contrast, the infection of B cells and Gr-1^+^ cells was associated with a significant increase in PD-L1 expression in comparison to naive cells (day 0) or to non-infected (gp70^-^) cells harvested at the same time point after infection (Fig 2B and 2C) This increase had two phases, it started early at 4 dpi but was most pronounced at 7–12 dpi corresponding with the expansion of cytotoxic CD8^+^ T cells at this later time point [26]. The histograms shown in Fig 2 indicate that infected (gp70^+^) CD19^+^ and Gr-1^+^ cells with high expression of PD-L1 were enriched during the infection process, most like because they survived CTL mediated killing (Fig 1E). Accordingly, the percentages of infected (gp70^+^) CD19^+^ (Fig 2B bars) and Gr-1^+^ cells (Fig 2C bars) expressing high levels of PD-L1 were increased as early as day 4 post infection reaching a plateau of more than 75% positive cells at day 7. Interestingly, the expression of PD-L1 on non-infected cells from infected mice was also enhanced during the second week of infection in comparison to cells from non-infected mice (day 0), most likely due to the inflammatory environment in the infected spleen.

To determine whether PD-L1 expression can influence survival of infected cells during acute FV infection, we took advantage of PD-L1 deficient mice [30]. PD-L1^-/-^ mice were infected and the percentages of FV infected surviving Ter119^+^ cells, B cells and Gr-1^+^ cells were calculated (Fig 1E; black bars). In comparison to wild type mice (Fig 1E; white bars) significantly less gp70^+^ B cells and Gr-1^+^ cells survived between 6 and 10 dpi in PD-L1 deficient mice during acute FV infection. Thus, the expression of PD-L1 on FV infected cells influenced the elimination of these CTL target cells.

### Retrovirus infection induces expression of PD-L1 on target cells *in vitro*

**Fig 3:**
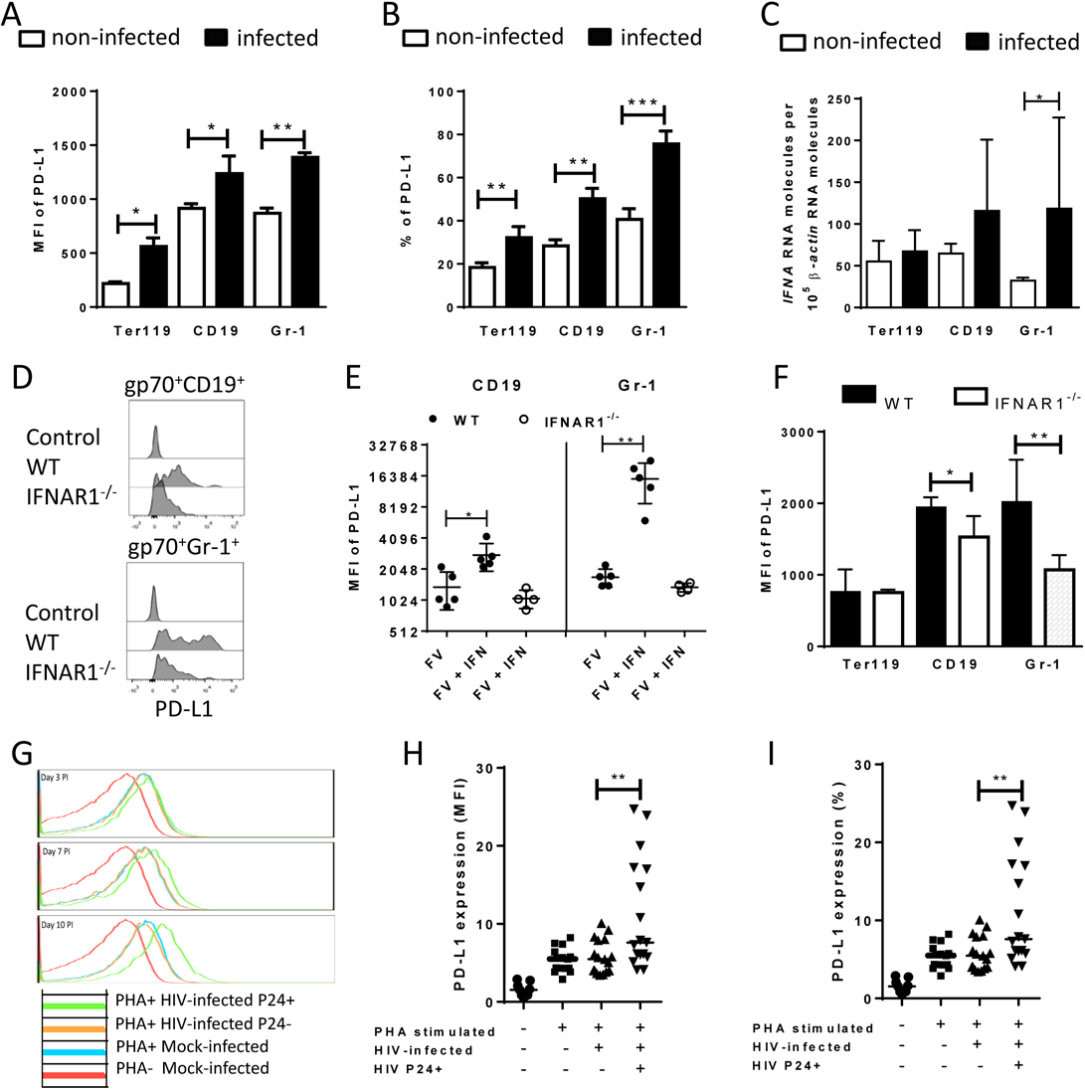
PD-L1 expression on cells infected in vitro with FV or HIV. Spleen cells were isolated from naive B6 mice and cultivated with F-MuLV infected *Mus Dunni* cells to infect mouse cells in vitro. Multi-parameter flow cytometry was used to determine PD-L1 expression (MFI) (**A**) and the percentage of PD-L1^high^ cells (**B**) in different target cell populations of FV. **C**. Ter119^+^, CD19^+^, and Gr-1^+^ cells were isolated from naïve wild type mice and were infected with F-MuLV *in vitro*. mRNA from infected and non-infected cells was isolated for real time PCR quantification of the IFNα mRNA expression. The numbers of IFNα mRNA copies in relation to 10^5^ copies of mRNA for *β*-actin is shown. Data was pooled from at least two independent experiments with similar results. Spleen cells were isolated from naїve wild type mice or from naïve IFNAR1^-/-^ mice and cultivated with F-MuLV infected *Mus Dunni* cells to infect mouse cells *in vitro*. Multi-parameter flow cytometry was used to determine PD-L1 expression (MFI) on infected CD19^+^ and Gr-1^+^ cells **(D**) and in the presence of IFNα (**E**) Data was pooled from at least two independent experiments with similar results. **F**. Multi-parameter flow cytometry was used to determine the expression of PD-L1 on the sur-face of gp70^+^Ter119^+^, gp70^+^CD19^+^, and gp70^+^Gr-1^+^ cells isolated from spleens of 6 day FV infected WT and IFNAR1^-/-^ mice. Data was pooled from two independent experiments with similar results. Multi-parameter flow cytometry was used to determine the expression of PD-L1 on the surface of human CD4^+^ T cells after HIV-1 infection. Representative histograms of PD-L1 expression on human CD4^+^ T cells non-stimulated and non-infected, stimulated *in vitro* with PHA and infected with HIV-1 or cells only stimulated with PHA are shown. The data is shown for day three, seven and ten after infection (**G**). Expression of PD-L1 on human CD4^+^ T cells (**H**) and the percentage of PD-L1^high^ CD4^+^ T cells (**I**) in populations of non-stimulated and non-infected, stimulated *in vitro* with PHA and infected with HIV-1 or cells only stimulated with PHA are shown at day ten after infection. Mean numbers plus SD from three independent experiments with similar results was shown. Differences between FV infected (gp70^+^) and FV non-infected (gp70^-^) mice cells were analyzed by an unpaired t-test. Differences between HIV infected (p24^+^) and HIV non-infected (p24^-^) CD4^+^ cells were analyzed by Mann-Whitney t test. Statistically significant differences between the groups are indicated in the figure (*p˂0.05, **p˂0.005).

It has been demonstrated that PD-L1 expression can be induced by inflammatory cytokines [31], however if retrovirus infection of target cells is associated with enhanced levels of PD-L1 expression was less well established. The initial upregulation of PD-L1 on FV-infected target cell suggest that virus infection might influence PD-L1 expression. To test this we performed *in vitro* infection experiments of spleen cells with FV (Fig 3A). Splenocytes from naïve mice were incubated with F-MuLV infected mouse fibroblasts *(M*. *Dunni*). Infection of spleen cells was determined by detection of gp70 expression on the cell surface of the three different cell populations of interest. Gp70 positivity was associated with a significantly enhanced expression of PD-L1 on infected Ter119^+^, CD19^+^, and Gr1^+^ cells in comparison to populations of non-infected cells (Fig 3A). The overall expression levels of PD-L1 were similar on B cells and Gr-1^+^ cells but much lower on Ter119^+^ erythroblasts. The percentage of PD-L1^high^ cells also significantly increased after FV infection (Fig 3B). Murine retroviruses have been reported to be sensed by TLR3 and TLR7 [32, 33] and this viral RNA recognition results in the induction of type I IFN responses [34]. Previous studies demonstrate that type I IFNs can stimulate the expression of PD-L1 on the surface of cells [35]. In order to determine whether FV infection induced transcription of IFNα *in vitro*, we compared the levels of mRNA by RT-PCR in infected versus non-infected Ter119^+^ cells, CD19^+^ and Gr-1^+^ cells. FV infection induced IFNα mRNA expression in CD19^+^ and Gr-1^+^ cells (Fig 3C). Thus, viral infection induces IFNα production *in vitro* and *in vivo* [34], but does IFNα influence the expression of PD-L1 on the surface of FV infected cells? To address this question spleen cells from wild type and IFN receptor deficient mice (IFNR1^-/-^) [36] were infected *in vitro*. FV infection more efficiently enhanced the PD-L1 expression level on the surface of gp70^+^ CD19^+^ cells and on gp70^+^ Gr-1^+^ cells from WT mice in comparison to IFNR1^-/-^ mice (Fig 3D). The expression of PD-L1 on the surface of FV infected CD19^+^ and Gr-1^+^ cells was significantly enhanced after treatment with exogenous IFNα (Fig 3E). This effect was not observed on FV infected cells from IFNR1^-/-^ mice. Moreover, gp70^+^ B cells and gp70^+^ Gr-1^+^ cells from FV infected IFNR1^-/-^ mice expressed significantly less PD-L1 than those cells from WT mice (Fig 3F). This data suggest that type I interferon signaling is involved in PD-L1 expression and that virus induced IFNα is at least one important regulator of PD-L1 expression on infected cells during the early phase of FV infection.

In order to show that human cells show enhanced expression of PD-L1 after infection with retrovirus as well, HIV infection of human CD4^+^ T cell was performed. It has previously been reported that the overall T cell compartment from HIV-infected patients expresses increased levels of PD-L1 [37], but this has not been attributed to virus infection on a single cell level. Therefore, we investigated the PD-L1 expression on CD4^+^ T cells from uninfected donors at day 3, 7 and 10 post HIV infection *in vitro*. Staining against intracellular p24 antigen was used to identify HIV infected cells. A representative histogram plot of a PD-L1 staining shows that PHA alone (which is needed to activate T cells so they become permissive to in vitro infection) increased PD-L1 expression but HIV infection further enhanced this expression during the course of infection (Fig 3G). Cumulative data of day 10 post infection from 8 donors shows that PD-L1 expression on HIV-infected CD4^+^ T cells (p24+) was significantly increased compared to p24^-^ cells from the same cultures (Fig 3H). Similar results were achieved by determining the percentages of infected CD4^+^ cells expressing high levels of PD-L1 (Fig 3I). These data suggest that HIV infection of CD4^+^ T cells also causes increased expression of PD-L1 that may help the virus to evade antiviral CTL responses.

### Up-regulation of PD-L1 on FV infected cells protect them from CTL killing

**Fig 4:**
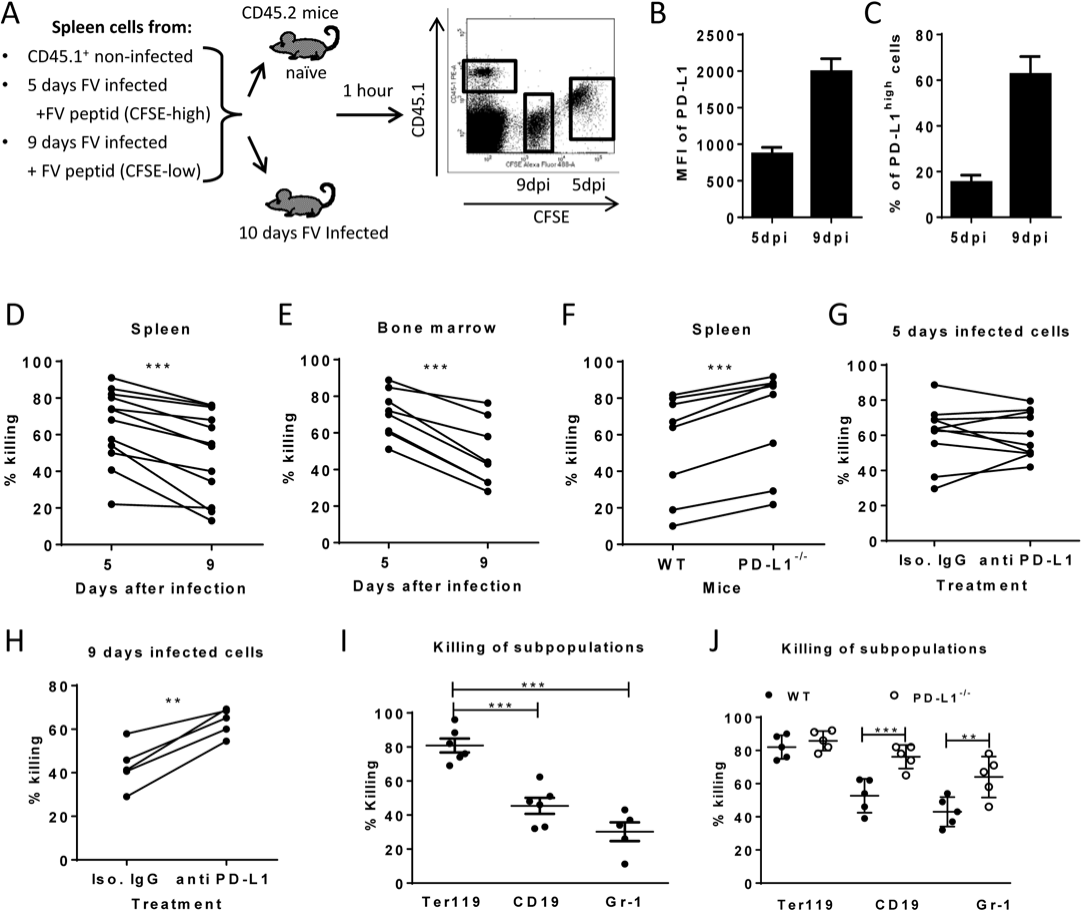
Cytotoxic activity of CTL against target cells expressing different levels of PD-L1. **A**. Splenocytes from FV infected mice were isolated at day 5 (PD-L1low) and day 9 (PD-L1high) post infection and used as target cells for an *in vivo* CTL assay. Therefore the cells were loaded with peptide and stained with different concentrations of CFSE. Spleen cells from naïve CD45-1 mice were used as control. Multi-parameter flow cytometry was used to compare the elimination of 5 day FV infected cells with cells from 9 day infected mice. MFI of PD-L1 expression (**B**) and percentage of cells expressing high level of PD-L1 (**C**) on surface of spleen cells isolated from 5 day and from 9 day FV infected mice. *In vivo* killing of target cells from 5 day and from 9 day infected mice in the spleens (**D**) and in the bone marrow (**E**) of 10 day FV infected mice. The data points received from the same recipient mouse were connected. **F**. *In vivo* killing of target cells from 9 day infected WT B6 and PD-L1 KO mice in the spleens of 10 day FV infected mice. The data points received from the same recipient mouse were connected. *In vivo* killing of target cells from 5 day (**G**) or cells from 9 day (**H**) FV infected B6 mice that were treated or non-treated *in vitro* with anti PD-L1 antibody before adoptive transfer into 10 day FV infected mice. The data points received from the same recipient mouse were connected. **I**. Elimination of Ter119^+^, CD19^+^ cells, and Gr-1^+^ cells transferred from 7 day infected mice in 10 day FV infected mice. (**J**). The elimination of target cells transferred from 9 day infected wild type and PD-L1^-/-^ mice in in FV infected recipients. Data was pooled from two to three independent experiments with similar results. Differences the elimination of target cell populations (D-H) was analyzed by paired t test. Differences elimination of subpopulations of target cells from 7 day infected mice (I, J) were analyzed by one-way ANOVA was used with a Tukey post-test. Statistically significant differences between the groups are indicated in the figure. (*p˂0.05, **p˂0.005, ***p˂0.0005).

Cytotoxic CD8^+^ T cells mediate elimination of infected target cells after day 6 of FV infection [26]. We hypothesize that cells with low-level expression of PD-L1 are more susceptible to CTL killing than cells expressing high levels of this molecule, which escape elimination by PD-1^high^ CTL and subsequently get enriched (Fig 2). In order to confirm this theory an *in vivo* CTL killing assay was performed (Fig 4A). This assay allows to differentially detect the elimination of different donor cell populations in the same mouse. Cells from 5 day infected mice (low level of PD-L1) and cells from mice infected for 9 days (high level of PD-L1) (Fig 4B and 4C) were loaded with FV D^b^GagL peptide [38, 39], mixed 1:1 and adoptively transferred as target cells for virus-specific CD8^+^ T cells into FV infected mice (Fig 4A). The elimination of both populations of target cells was simultaneously analyzed in the same infected donor mouse (connected dots in Fig 4). This analysis was performed in the spleen (Fig 4D) and bone marrow (Fig 4E), because those are the organs with the highest FV loads and the strongest CTL activity [26]. In both organs elimination of target cells from donor mice was detected. However, the killing of splenocytes from 5 day infected mice (PD-L1low) was significantly higher in comparison to splenocytes from 9 day infected mice (PD-L1high). This confirms that enhanced expression of inhibitory ligands protected infected target cells from CTL killing.

**Fig 5:**
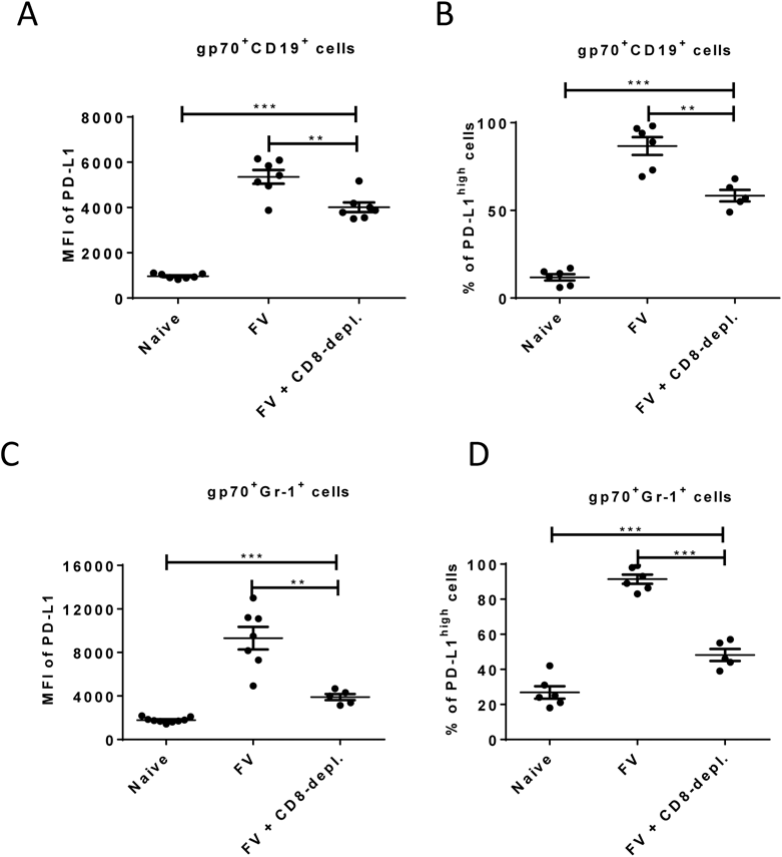
Expression of PD-L1 on FV infected cells from CD8^+^ T cells depleted mice. C57BL/6 mice were infected with FV and treated with anti-mouse CD8 antibody. The spleen cells were isolated at day 10 after infection. Multi-parameter flow cytometry was used to compare the expression of PD-L1 on the cell surface of CD19^+^ cells (A) and Gr-1^+^ cells (C) and the percentage of infected (gp70^+^) CD19^+^ (B) and Gr-1^+^ (D) cells expressing high levels of PD-L1. Data was pooled from three independent experiments with similar results. Differences in PD-L1 expression on CD19^+^ cells or Gr-1^+^ cells from naïve mice and gp70^+^ CD19^+^ cells or gp70^+^ Gr-1^+^ cells from mice FV infected and FV infected and CD8^+^ T cells depleted mice were analyzed by one-way ANOVA was used with a Tukey post-test. Statistically significant differences between the groups are indicated in the figure (*p˂0.05, **p˂0.005, ***p˂0.0005).

In order to directly demonstrate the involvement of PD-L1 in the escape of target cells from CTL killing *in vivo*, two killing experiments with an impaired PD-L1 function on target cells were performed. Splenocytes from 9 day infected wild type mice or PD-L1 knockout mice were used as targets for CTL *in vivo* (Fig 4F). As expected, PD-L1 deficient target cells were more efficiently eliminated than wild type cells. Another modification of the *in vivo* CTL assay was performed to directly show the influence of PD-L1 on the efficacy of target cell killing. Half of the FV D^b^GagL peptide loaded spleen cells from 9 day infected mice (PD-L1^high^) were treated with blocking anti-PD-L1 antibodies and the other half with an isotype control antibody prior to their adoptive transfer into infected donor mice (Fig 4H). In all recipient mice splenocytes treated with anti-PD-L1 antibodies were killed significantly better than the cells with the control antibody. If the same experiment was performed with spleen cells from 5 day infected mice that express only low levels of PD-L1 either slight enhancement or reduction of target cell elimination after treatment with anti-PD-L1 antibodies was observed (Fig 4G). If a 10% increase of target cell killing in the population of anti-PD-L1 treated target cells was considered as significant enhancement compared to the isotype control, then only 2 out of 10 animals showed that in the group of mice receiving 5 day infected cells (Fig 5G), whereas all mice (5 out of 5) showed this in the 9 day group (Fig 5H). These different numbers were analyzed with the Mann-Whitney-Rank test, which indicates a significant difference between the groups with a p-value of 0,0014.

The data clearly indicate that PD-1 signaling is influencing the efficacy of CTL killing during acute FV infection.

To analyze whether the differences in PD-L1 expression between the Ter119^+^, CD19^+^ and Gr-1^+^ infected target cell populations and their differences in survival during acute FV infection correlated with CTL mediated elimination, we determined the killing of specific target cell subsets from 7 day infected mice in our *in vivo* CTL assay (Fig 4I). FV-specific CD8^+^ T cells most efficiently eliminated the Ter119^+^ cells (PD-L1^low^, Fig 2A), whereas killing of B cells (PD-L1^high^, Fig 2B) and Gr-1^+^ cells (PD-L1^high^, Fig 2C) was significantly reduced to half or even less than half of the Ter119^+^ cell killing. Thus, the PD-L1 expression levels on the different populations of infected target cells clearly correlated with the ability of virus-specific CTL to kill these cells during acute FV infection. In order to show the functional effect of the PD-L1 signaling on the CTL killing of different subpopulations, FV D^b^GagL peptide loaded spleen cells from 9 day infected wild type or PD-L1 knockout mice were used as targets for the killing assay (Fig 4J). When PD-L1 was absent the large difference in target cell elimination between Ter119^+^ cell and CD19^+^/Gr-1^+^ was gone as infected B cells and myeloid cells from PD-L1 knockout mice were significantly better eliminated than those from wild type mice. Thus, the lack of PD-L1 on the surface of infected target cells resulted in an enhanced susceptibility to CD8 T cell mediated killing.

### Enrichment of infected PD-L1^high^ cells was mediated by CTL

Enhancement of PD-L1 expression on infected CD19^+^ and Gr-1^+^ cells after day 6 post FV infection occurs concomitant with the expansion of virus-specific CTL (Fig 2 and [26]). This suggests that CTL preferentially eliminate PD-L1^low^ target cells, but PD-L1^high^ cells evade killing and become enriched over time. In order to test this FV infected mice were depleted for CD8^+^ T cells and the expression of PD-L1 on infected B cells and myeloid cells was analyzed.

As previously shown (Fig 2), the expression of PD-L1 is significantly increased on infected CD19^+^ and Gr-1^+^ cells at 10 days post infection suggesting a preferential survival of PD-L1^high^ cells (Fig 1E). However, the level of PD-L1 expression and the percentages of cells expressing high levels of PD-L1 on infected B cells (Fig 5A and 5B) and on infected myeloid cells (Fig 5C and 5D) was significantly reduced in mice depleted of CD8^+^ T cells. During previous studies it was observed that mice without CD8^+^ effector T cells were unable to efficiently control the replication of FV, since cytotoxicity is the main functional property of FV-specific CD8^+^ T cells [40]. Thus, the low mean fluorescence intensity values in mice deficient for CD8^+^ T cells reflects the lack of cytotoxic T cell pressure on the infected target cells or the effect of proinflammatory cytokines produced by effector CD8^+^ T cells. However, the PD-L1 expression levels on infected CD19^+^ and Gr-1^+^ cells from CD8^+^ depleted mice were still significantly higher than those of non-infected cells of the same population. The data demonstrate the enrichment of PD-L1^high^ target cells by CTL activity but also imply that the infection of the cells itself, most likely by production of type I IFN, induced up-regulation of PD-L1.

### High expression of PD-L1 on infected target cells suppresses the functionality of CD8^+^ T cells

**Fig 6:**
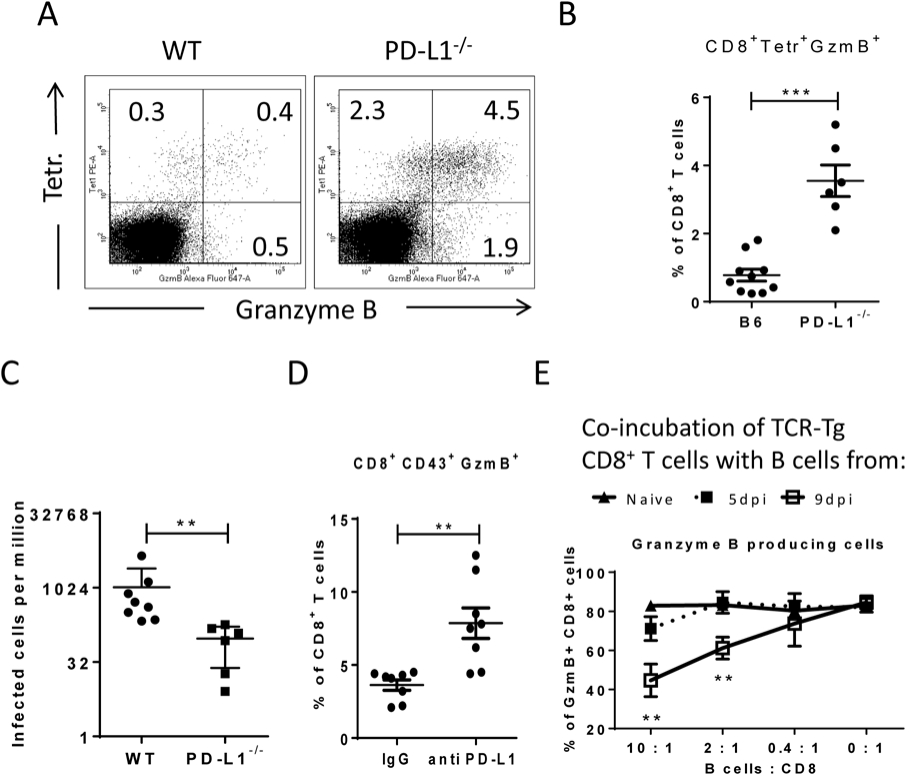
Suppression of CD8^+^ T cell function by PD-L1 expressing target cells. C57BL/6 and PD-L1^-/-^ mice were infected with FV. Multi-parameter flow cytometry was used to compare the populations of FV gag tetramer positive CD8^+^ T cells at 8 days after infection. **A**. Representative dot plots gated on CD3^+^CD8^+^ T cells. Tetramer^+^ cells were stained for granzyme B expression. **B**. The percentage of CD8^+^Tetramer^+^Granzyme B^+^ cells per one million nucleated spleen cells is shown. **C**. The numbers of infectious cells in the spleens of 10 day infected wild type and PD-L1^-/-^ mice. **D**. C57BL/6 mice were infected with FV and treated with anti PD-L1 antibody or with mice IgG as a control group. Spleen cells were isolated at day ten after infection. The number of CD8^+^CD43^+^GzmB^+^ cells per one million nucleated cells was determined by flow cytometry. **E**. Naïve CD8^+^ T cells from FV-specific TCR transgenic mice were stimulated with FV peptide loaded DCs and incubated with different numbers of B cells from naive, 5 day infected or 9 day infected mice. The production of granzyme B in CD8^+^ T cells was analyzed after 48h of co-incubation. Data was pooled from three independent experiments with similar results. Differences were analyzed by paired t-test. Statistically significant differences between the groups are indicated in the figure (**p˂0.005, ***p˂0.0005).

Virus-specific CD8^+^ T cells form tight contacts with infected target cells called cytotoxic synapses. Binding of virus-specific PD-1^high^ CTLs to infected targets with high expression of PD-L1 may therefore have functional consequences for the effector CD8^+^ T cell. In order to analyze this, we infected PD-L1 knockout mice and compared their CTL response with wild type animals (Fig 6A and 6B). The absence of PD-L1 resulted in enhanced expansion of virus-specific CD8^+^ T cells and augmented production of the cytotoxic molecule granzyme B by FV-specific (tetramer^+^) CD8^+^ T cells. The expanded effector CD8^+^ T cells efficiently controlled FV infection in PD-L1 knockout mice (Fig 6C). Similar results were obtained from 10 day infected wild type mice treated once at day 7 with anti-PD-L1 blocking antibody (Fig 6D). When binding of PD-L1 to the PD-1 receptor was blocked by the antibody more granzyme B was produced by activated CD43^+^CD8^+^ T cells. These *ex vivo* data demonstrate the regulatory role of PD-L1 on the functionality of CD8^+^ T cells during acute FV infection.

In order to show that target cells with enhanced expression of PD-L1 potently inhibit the cytotoxic functions of CD8^+^ T cells the following *in vitro* experiment was performed (Fig 6E). Naïve CD8^+^ T cells from TCR transgenic mice [39, 41] specific for the FV GagL immunodominant epitope were stimulated with DC loaded with respective FV peptide (FV GagL CTL epitope) to induce activation and proliferation of the CD8^+^ T cells. We then added different numbers of B cells isolated either from naïve mice or mice infected with FV for 5 (PD-L1^low^) or 9 (PD-L1^high^) days. The stimulation of the FV GagL-specific CD8^+^ T cells with their cognate antigen resulted in cell proliferation and more than 80% of the CD8^+^ T cells started to produce granzyme B. Adding increasing numbers of naïve B cells to these cultures did not change T cell proliferation or granzyme B production (Fig 6D). Also FV infected B cells expressing low levels of PD-L1 (from 5 day infected mice) did not change proliferation or function of the CD8^+^ T cells. However, the production of the cytotoxic molecule granzyme B in T cells was significantly reduced after incubation with PD-L1^high^ B cells from 9 day infected mice. The magnitude of the suppressive effect of PD-L1^high^ B cells was dependent on B cell numbers. The current data from *in vivo* and *in vitro* experiments prove that infected target cells expressing high levels of PD-L1 suppress the proliferation and functionality of anti-viral CD8^+^ T cells. In FV infected mice this effect starts at a late phase of acute infection (9 dpi) and results in immune escape of infected cells.

## Discussion

The functionality of antigen-specific cytotoxic T cells is regulated by a number of positive and negative signaling pathways. Inhibitory signals are crucial for the development of peripheral tolerance and for preventing autoimmune disorders. The PD-1 inhibitory receptor also plays a key role in the development of exhaustion of virus-specific CD8^+^ T cells during numerous chronic infections [42]. Blocking of PD-1 signaling by antibody treatment partially reconstitutes the functionality of CD8^+^ T cells during chronic infection and can even result in elimination of viruses [15]. However, the kinetic of T cell exhaustion and the involvement of the PD-1 ligand PD-L1 is less well established in many virus infections. PD-1 and other inhibitory receptors are often up-regulated on virus-specific CD8^+^ T cells very early after infection and can even be used as activation markers during several acute viral infections. Although, the impact of PD-1 expression on T cells during acute infections is quite contradictory. Some studies suggested that the expression of PD-1 was associated with T cell dysfunction during initial infection [12, 43, 44]. In other infection models PD-1 expression did not induce exhaustion of virus-specific CD8^+^ T cells during acute infection [10, 14, 45]. Even studies using the same infection model reported contradictory findings. For example, Takamura et al. claimed that PD-1^high^ CD8^+^ T cells are prematurely exhausted during acute FV infection of mice and play only a limited role in virus control [46]. However, in a subsequent study we could clearly demonstrate that FV-specific PD-1^high^ CTL efficiently killed virus infected target cells and were critical for reducing acute viral loads [14]. During initial FV infection PD-1 is an activation rather than an exhaustion marker for CD8^+^ T cells. However, after several days of acute FV infection (>9dpi) T cells start to become dysfunctional and signaling through PD-1 receptor can contribute to this exhaustion. Our current study provides an explanation for our previous findings. The critical factor that determines whether or not PD-1 expression is resulting in T cell exhaustion is the expression of the ligand PD-L1 on cells that have close contact to CD8^+^ T cells. During FV infection of resistant mice the main target cell population of the virus are Ter119^+^ cells but for an unknown reason the expression of PD-L1 on these cells was not changed after infection. Consequently, these target cells were almost completely eliminated by virus-specific CTL during the acute infection. In contrast, in the other two important target cell populations, B cells and myeloid cells, FV infection resulted in a significant up-regulation of PD-L1 during the initial phase of infection (4 dpi, Fig 2). This expression of inhibitory receptors protected infected cells from cytotoxic killing and thus, it is not surprising that B cells and myeloid cells ultimately form the viral reservoir in chronic FV infection [23]. The immune escape of infected PD-L1^high^ cells enriched these cells during the late phase of acute infection (>7 dpi), whereas infected PD-L1^low^ CD19^+^ and Gr-1^+^ cells were eliminated (Fig 4I). Despite killing virus-specific CD8^+^ T cells also produce proinflammatory cytokines, like TNFα and IFNγ. These cytokines were shown to enhance the expression of PD-L1 in infected organs [31]. Thus, the cytotoxic activity of CD8^+^ T cells and the secretion of cytokines by these cells might contribute to the accumulation of infected cells with enhanced PD-L1 expression during the establishment of a chronic infection.

During the late phase of acute FV infection (>9dpi) many infected cells, which express high levels of PD-L1, are left as targets for activated PD-1^+^ CTL. The multiple inhibitory signals from these PD-L1^high^ targets then induce the functional exhaustion of virus-specific CD8^+^ T cells (Fig 6E). Similar findings were obtained by *in vivo* imaging of CD8^+^ T cell responses against LCMV [47]. The enhanced expression of PD-L1 on LCMV infected cells abrogated the migration of virus-specific CD8^+^ T cells and resulted in formation of long-lasting immunological synapses. The outcome of this prolonged CTL target cell interaction was a PD-L1 dependent migration arrest of antigen-specific CD8^+^ T cells and a decreased production of pro-inflammatory cytokines by these cells. Also in other studies of LCMV infection [48, 49] an enhanced PD-L1 expression on infected cells was observed and the suppressive effect of this ligand on the functionality of virus-specific CD8^+^ T cells was demonstrated. Thus, PD-L1 on infected cells directly suppresses the functionality of virus-specific CD8^+^ T cells.

The first moderate but significant enhancement of PD-L1 expression was observed directly after infection, which suggests that this enhancement was induced by the virus. The FV infection of cells *in vitro* (Fig 3C) and *in vivo* [50] induces the transcription of IFNα mRNA, which can up-regulate PD-L1 expression on infected cells. Thus, the virus-induced production of IFNα is one possible mechanism regulating the expression of PD-L1 on the surface of infected target cells. Similar effects as those of FV infection on PD-L1 expression were also found for the infection of human CD4^+^ T cells with HIV-1. Such an enhanced expression of PD-L1 was previously observed in *in vitro* HIV-1 infected human macrophages [20]. The induction of PD-L1 expression on HIV-1 infected cells is mediated by the HIV-1 Tat protein [51]. An enhanced expression of PD-L1 on the surface of infected cells was also observed after infection with MCMV [52], influenza virus [53], Theiler's murine encephalomyelitis virus [54], and human rhinovirus [55]. Obviously, PD-L1 expression on the surface of infected target cells can be regulated by different viruses and infected cells expressing PD-L1 acquire immunoregulatory properties.

The expression of PD-L1 was observed in different types of tumors [56]. The suppressive effect of this molecule on the T cell antitumor immunity was also demonstrated. Moreover, the prevention of PD-1 signaling after treatment with anti-PD-1 or anti-PD-L1 therapeutic antibody shows the effective elimination of tumors in the experimental models as well as in clinical trials [15]. The mechanisms regulating the expression of PD-L1 on tumor cells are not completely understood, but the recent study shows that CD8^+^ T cells induce the expression of PD-L1 on melanoma tumor cells [57]. Based on current data, we propose that during tumor elimination CTLs preferentially kill cells with a low expression of PD-L1, whereas cells with a high expression of PD-L1 seem to escape from this elimination and subsequently accumulate.

Thus the provided data concerning PD-L1 expression on infected cells and regulation of CTL functionality may be helpful information for the development of new therapeutic approaches against chronic viral infections and cancer.

## Materials and Methods

### Ethics statement

Animal experiments were performed in strict accordance with the German regulations of the Society for Laboratory Animal Science (GV-SOLAS) and the European Health Law of the Federation of Laboratory Animal Science Associations (FELASA). The protocol was approved by the North Rhine-Westphalia State Agency for Nature, Environment and Consumer Protection (LANUV) (Permit number: G 1252/10 and G 1193/11). All efforts were made to minimize suffering.

### Mice

Inbred C57BL/6 (B6) mice were maintained under pathogen free conditions. Experiments were performed using C57BL/6 (B6) mice. The relevant FV resistance genotype of B6 mice is H-2^b/b^, Fv1^b/b^, Fv2^r/r^, Rfv3^r/r^. The B6 mice were obtained from Charles River Laboratories. B6-background PD-L1 KO (B7-H1-KO) mice were originally generated by L.C. [30]. B6.SJL-Ptprca Pep3b/BoyJ (CD45.1) B6-background mice were obtained from Charles River Laboratories. IFNAR knockout mice [36] were backcrossed more than 10 times on a C57BL/6 background. DbGagL TCR tg mice were on a C57BL/6 background and more than 90% of the CD8+ T cells contained a TCR specific for the DbGagL FV epitope [39, 41]. All mice were females 8–16 weeks of age at the beginning of the experiments.

### Virus and viral infection

The FV stock used in these experiments was a FV complex containing B-tropic Friend murine leukemia helper virus (F-MuLV) and polycythemia-inducing spleen focus-forming virus free of lactate dehydrogenase-elevating virus [58, 59]. The stock was prepared as a 10% spleen cell homogenate from BALB/c mice infected 14 days previously with 3 000 spleen focus-forming units of non-cloned virus stock. Experimental mice were injected intravenously with 0.3ml of PBS containing 20 000 spleen focus-forming units of FV.

10 MOI of F-MuLV was used for in vitro infection of *Mus Dunni* cells. 24 hrs infected *Mus Dunni* cells were incubated with spleen cells from naïve CD45.1 mice for 48h; spleen cells were then isolated for flow cytometry analysis. Alternatively naïve spleen cells from wild type mice or from IFNAR^-/-^ mice were incubated for 48h with non-infected or F-MuLV infected *Mus Dunni* cells with the addition of 1000U Universal type I Interferon (PBL Assay).

### Phenotypic analysis of PD-L1 during HIV infection of human CD4^+^ T cells

Blood was collected from healthy donors under an approved Institutional Review Board protocol. Peripheral blood mononuclear cells (PBMC) were purified by Ficoll-Hypaque density gradient separation as previously described [60]. CD4^+^ T cells were isolated by positive selection using magnetic bead separation according to the manufacturer’s protocol (Miltenyi Biotec, Auburn, CA). 1x10^7^ CD4^+^ T cells were cultured in RPMI 1640 (Invitrogen, Gaithersburg, MD) containing 10% fetal bovine serum (Hyclone, Logan, UT) and 1% Pen-Strep-Glut (Invitrogen) in a 6-well plate. Cells were pretreated with polybrene (4ug/ml) for 30 minutes to improve viral infection. After two washes, cells were resuspended in complete medium in the absence or presence of 2 μg/ml PHA (Sigma). Cells were cultured overnight in the absence or presence of cell free NL4.3 HIV virus (MOI 0.1) at 37°C in a humidified 5% CO_2_ atmosphere. The following day, cells were washed twice and resuspended at 2x10^6^ cells per milliliter culture media. Cells were collected at days 3, 7 and 10 and were stained with fluorescent antibodies against CD3 (OKT3, BioLegend), CD4 (OKT4, BioLegend), CD8 (3B5, Invitrogen), PD-1 (MIH4, BD Pharmingen) and PD-L1 (MIH1, eBioscience) before fixation. The cells were then permeabilized (BD Cytoperm) and stained for intracellular p24 antigen (KC57-RD1, Beckman Coulter). The cells were analyzed by flow cytometry, and CD3^+^CD8^-^ cells were gated to identify p24 positive and negative populations and the expression levels of PD-L1 was measured on both populations.

### Cell surface and intracellular staining by flow cytometry

Cell surface staining was performed using Becton Dickinson or eBioscience reagents. Following antibodies were used: anti-CD3, anti-CD4 (RM4-5), anti-CD8 (53–6.7), anti-CD19 (ID3), anti-CD43 (1B11), anti-CD45.1 (A20), anti-Gr-1 (RB6-8C5), anti-PD-L1 (MIH-5), and anti-Ter119 (Ter-119). Dead cells (propidium iodide positive) were excluded from analyses. Intracellular granzyme B (monoclonal anti-human granzyme B (GB11), (Invitrogen, Darmstadt, Germany) staining was performed as described [26]. FV protein gp70 expressing cells were detected by labeling with antibody 720 (Ab720) [25]. Ab720 were isolated from hybridoma supernatant and conjugated with Alexa Fluor 647 according to manufacturer’s protocol (Molecular Probes). Data were acquired on a LSR II flow cytometer (Becton Dickinson) from 200,000–300,000 lymphocyte-gated events per sample. Analyses were done using FlowJo (Treestar) and FACSDiva software (Becton Dickinson). The quantity of survived cells at day 10 was calculated by determining the portion of cells detectible at day 10 after infection from the numbers of infected cells at the peak of FV infection at day 6. These quantities were calculated for every cell population separately.

### Tetramers and tetramer staining

For the detection of D^b^-GagL-specific CD8^+^ T cells, spleen cells were stained with PE labelled MHC class I H2-D^b^ (Beckman Coulter, Marseille, France) tetramers specific for FV GagL peptide [38, 39] as described previously (Zelinskyy et al., 2009).

### *In vivo* cytotoxicity assay

The *in vivo* CTL assay described by Barber et al. [61] was modified to measure cytotoxicity in FV-infected mice (Fig 4A). Splenocytes from mice infected for 5 or 9 days were loaded with 1–5 μM D^b^GagL peptide. The peptide loaded cells were stained with 4nM or 200 nM of CFSE (Molecular Probes). As a reference, splenocytes isolated from naïve CD45.1 mice were used. Splenocytes (1x10^7^ cells of each population) were transferred i.v. into naïve or 10 day FV-infected mice. One hour after adoptive transfer, spleens and bone marrows from recipient mice were harvested and cell suspensions were prepared. Cell suspensions were stained with anti CD45.1 antibody and measured by LSR II. Donor cells were distinguished from recipient cells and from one another based on different CFSE intensities and on expression of CD45.1 (Fig 4A, dot plot). The percentage of killing of each population of FV pulsed cells was calculated as follows: 100 - ([(% peptide pulsed in infected / % CD45.1+ unpulsed in infected) / (% peptide pulsed in uninfected / % CD45.1+ unpulsed in uninfected)] x 100). [38, 39].

In order to show the direct role of PD-L1 for elimination of target cells the *in vivo* cytotoxicity assay was modified. Cells from 9 day FV infected PD-L1 KO mice or cells from 9 day infected C57BL/6 mice in vitro treated with anti PD-L1 antibody (10F.9G2) (BioXCell) were used instead of cells from 5 day infected mice in above described in vivo cytotoxicity assay. The remaining steps of assays were performed as described above. A control experiment was performed with target cells from 5 day infected C57BL/6 mice treated with anti PD-L1 antibody (10F.9G2; BioXCell) and injected together with non-treated cells from 5 day infected mice and with a reference population from CD45.1 mice.

### PD-L1 blockade and lymphocyte depletion

C57BL/6 mice were infected with FV. 250 μg of anti PD-L1 antibody (10F.9G2) (BioXCell) or control rat IgG antibody (BioXCell) was administered i.p. at the time of infection and every other day for a total of 5 injections. CD8^+^ T cell depletion was started simultaneously with FV infection. Mice were inoculated every other day for 5 times intraperitoneally (i.p.) with 0.5 mL of supernatant fluid obtained from hybridoma cell culture 169.4 producing CD8a-specific monoclonal antibody [62]. The treatment depleted more than 95% of the CD8^+^ cells in the spleen (at 10 days post infection).

### *In vitro* suppression assay

To examine the influence of PD-L1high cells in vitro on both proliferation and function of CD8^+^ T cells, we modified a standard in vitro immunosuppression assay as described for the characterization of the suppressive function of regulatory T cells [63]. Mice bone marrow derived dendritic cells were generated as previously described (Balcow et al.) and incubated with DbGagL peptide (5 μg/ml) (28) in RPMI (Life Technologies) containing 10% normal mouse serum for 60 min at 37°C. FV-specific TCR Tg CD8^+^ T cells were isolated from spleens of DbGagL TCR Tg mice [39, 41] by positive selection using magnetic bead separation according to the manufacturer’s protocol (Miltenyi Biotec), and then labeled with 5 μM CFSE (Molecular Probes). B cells (CD19^+^) were isolated from spleens of naïve, 5 days, and 9 days FV infected B6 mice by positive selection using magnetic bead separation according to the manufacturer’s protocol (Miltenyi Biotec). Purities of all cell populations were >94%. For the induction of T cell proliferation, 1 × 10^5^ of peptide-pulsed DCs and 5 x 10^5^ TCR Tg CD8^+^ T cells per well were cultured on a flat-bottom 96-well plate in AIM-V (Life Technologies) containing 10% FBS, 2 mM L-glutamine, 50 μM 2-ME and 100 U/ml each penicillin and streptomycin at 37°C with 5% CO2. B cells were added to cultures simultaneously with CD8^+^ T cells at a 10:1, 2:1, and 0,4:1 ratio of B cells to CD8^+^ target cells. After 48 h cultivation cells were then stained for CD8, fixed, permeabilized, and stained for intracellular granzyme B as described above.

### RNA isolation and real time-PCR

Total RNA of 500 000 cells was isolated using TRIzol reagent (Life Technologies) and Pure Link RNA Micro Kit (Life Technologies). Isolated RNA was dissolved in RNase-free water and stored at -80°C. Total RNA concentration was determined by using NanoDrop 2000c spectrometer (Thermo Scientific, Wilmington, DE). Real time-PCR analysis for the quantification of IFN-α mRNA was performed using Power SYBR Green RT-PCR kit (Life Technologies) Primer sequences (Biomers) were as follows: 5’-atggctaggctctgtgctttcct-3’, 5’-agggctctccagacttctgctctg-3’. The absolute mRNA copy numbers were determined by using StepOne Software v2.3 (Life Technologies).

### Statistical analysis

Statistics comparing the two groups were done using the unpaired non-parametric t test or Mann-Whitney t test. Statistics comparing the elimination of the two groups of target cells *in vivo* were done using the paired non-parametric t test. When more than two groups were compared, a one-way ANOVA was used with a Tukey post-test. (GraphPad Prism software; GraphPad Software Inc., San Diego, USA).
