# Correction: PD-L1 Expression on Retrovirus-Infected Cells Mediates Immune Escape from CD8+ T Cell Killing

**DOI:** 10.1371/journal.ppat.1005364

**Published:** 2015-12-18

**Authors:** Ilseyar Akhmetzyanova, Malgorzata Drabczyk, C. Preston Neff, Kathrin Gibbert, Kirsten K. Dietze, Tanja Werner, Jia Liu, Lieping Chen, Karl S. Lang, Brent E. Palmer, Ulf Dittmer, Gennadiy Zelinskyy

The authors would like to correct panel H in Fig 3 to show the correct MFI values of PD-L1 expression on human CD4+ T cells. The error occurred during preparation of the figure for manuscript revision. The percentages of infected CD4 cells expressing PD-L1 were accidentally duplicated from panel I in Fig 3. Please see the corrected version of Fig 3 here.

**Fig 3:**
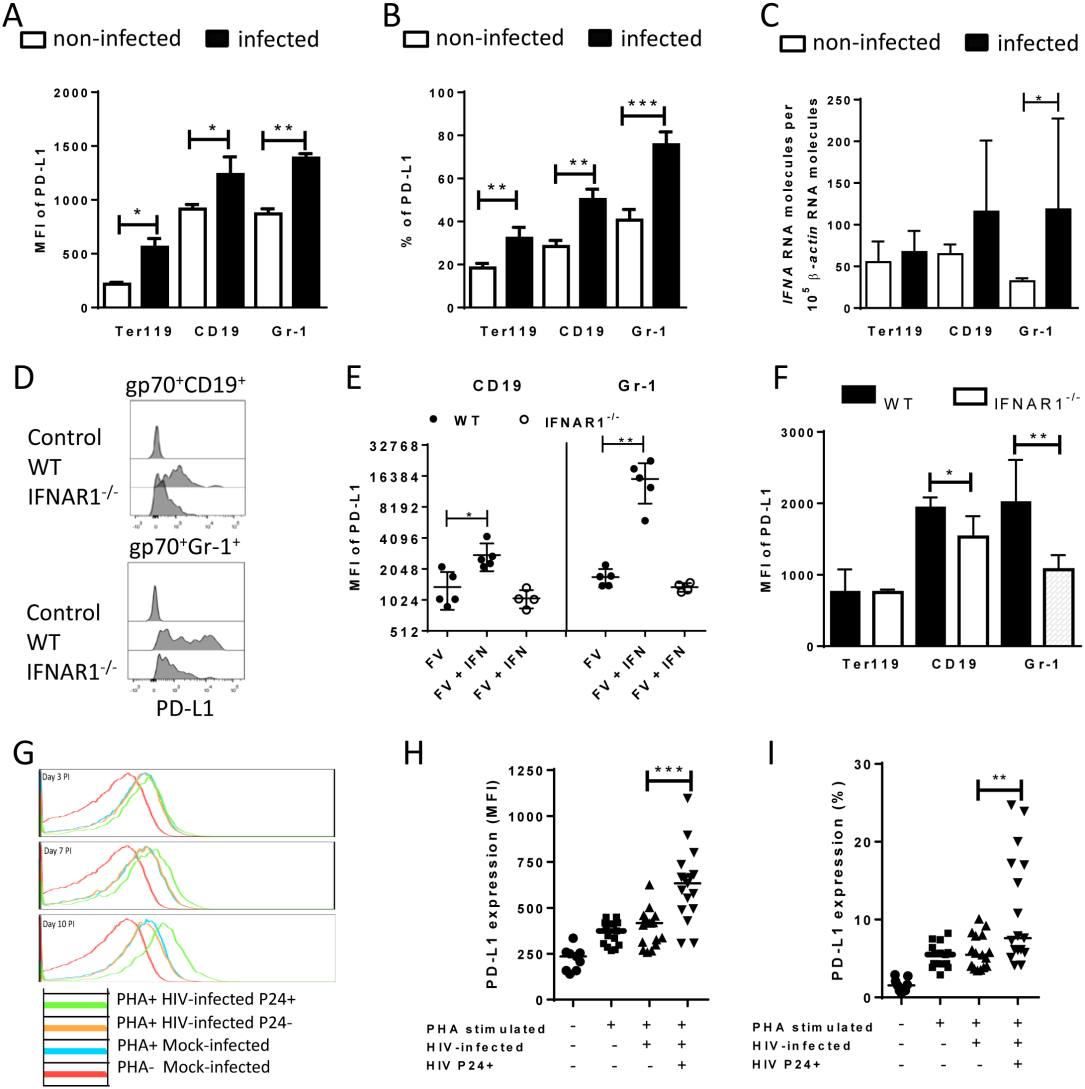
PD-L1 expression on cells infected in vitro with FV or HIV. Spleen cells were isolated from naive B6 mice and cultivated with F-MuLV infected *Mus Dunni* cells to infect mouse cells in vitro. Multi-parameter flow cytometry was used to determine PD-L1 expression (MFI) (**A**) and the percentage of PD-L1^high^ cells (**B**) in different target cell populations of FV. **C**. Ter119^+^, CD19^+^, and Gr-1^+^ cells were isolated from naïve wild type mice and were infected with F-MuLV *in vitro*. mRNA from infected and non-infected cells was isolated for real time PCR quantification of the IFNα mRNA expression. The numbers of IFNα mRNA copies in relation to 10^5^ copies of mRNA for *β*-actin is shown. Data was pooled from at least two independent experiments with similar results. Spleen cells were isolated from naїve wild type mice or from naïve IFNAR1^-/-^ mice and cultivated with F-MuLV infected *Mus Dunni* cells to infect mouse cells *in vitro*. Multi-parameter flow cytometry was used to determine PD-L1 expression (MFI) on infected CD19^+^ and Gr-1^+^ cells **(D**) and in the presence of IFNα (**E**) Data was pooled from at least two independent experiments with similar results. **F**. Multi-parameter flow cytometry was used to determine the expression of PD-L1 on the sur-face of gp70^+^Ter119^+^, gp70^+^CD19^+^, and gp70^+^Gr-1^+^ cells isolated from spleens of 6 day FV infected WT and IFNAR1^-/-^ mice. Data was pooled from two independent experiments with similar results. Multi-parameter flow cytometry was used to determine the expression of PD-L1 on the surface of human CD4^+^ T cells after HIV-1 infection. Representative histograms of PD-L1 expression on human CD4^+^ T cells non-stimulated and non-infected, stimulated *in vitro* with PHA and infected with HIV-1 or cells only stimulated with PHA are shown. The data is shown for day three, seven and ten after infection (**G**). Expression of PD-L1 on human CD4^+^ T cells (**H**) and the percentage of PD-L1^high^ CD4^+^ T cells (**I**) in populations of non-stimulated and non-infected, stimulated *in vitro* with PHA and infected with HIV-1 or cells only stimulated with PHA are shown at day ten after infection. Mean numbers plus SD from three independent experiments with similar results was shown. Differences between FV infected (gp70^+^) and FV non-infected (gp70^-^) mice cells were analyzed by an unpaired t-test. Differences between HIV infected (p24^+^) and HIV non-infected (p24^-^) CD4^+^ cells were analyzed by Mann-Whitney t test. Statistically significant differences between the groups are indicated in the figure (*p˂0.05, **p˂0.005).
